# Krt6a-Positive Mammary Epithelial Progenitors Are Not at Increased Vulnerability to Tumorigenesis Initiated by ErbB2

**DOI:** 10.1371/journal.pone.0117239

**Published:** 2015-01-30

**Authors:** Kimberly R. Holloway, Vidya C. Sinha, Michael J. Toneff, Wen Bu, Susan G. Hilsenbeck, Yi Li

**Affiliations:** 1 Lester & Sue Smith Breast Center, Baylor College of Medicine, Houston, TX, United States of America; 2 Department of Molecular and Cellular Biology, Baylor College of Medicine, Houston, TX, United States of America; 3 Department of Molecular Virology and Microbiology, Baylor College of Medicine, Houston, TX, United States of America; University of North Carolina School of Medicine, UNITED STATES

## Abstract

While most breast cancers are thought to arise from the luminal layer of the breast tissue, it remains unclear which specific cells in the luminal layer are the cells of origin of breast cancer. We have previously reported that WAP-positive luminal epithelial cells are at increased susceptibility to tumor initiation by ErbB2 compared to the bulk population, while the mammary cells with canonical Wnt signaling activity fail to evolve into tumors upon ErbB2 activation. Here, we used retrovirus to introduce ErbB2 into the Krt6a-positive mammary progenitor subset of the luminal epithelium and, for comparison, into the mammary luminal epithelium indiscriminately. Tumors developed from both groups of cells with a similar latency. These data indicate that the Krt6a-positive subset of mammary epithelial cells can be induced to form cancer by ErbB2 but it is not more susceptible to tumorigenesis initiated by ErbB2 than the bulk population of the luminal epithelium.

## Introduction

The breast epithelial compartment is organized like a branching tree with large ducts, ductules, and alveoli, the latter of which can produce milk during late pregnancy and lactation. These ducts and alveoli are lined with an inner layer of luminal epithelial cells and an outer layer of myoepithelial cells. Most breast cancers are thought to arise from the luminal epithelial layer [1]. Cells in this luminal epithelial layer are heterogeneous in differentiation and expression of genes important in breast development and function. For example, cells expressing CD61 (β3 integrin) are less differentiated than others, while those that produce estrogen receptor (ER) appear to be more differentiated [2,3]. Whey acidic protein (WAP) is made by a subset of differentiated luminal epithelial cells especially those that line alveoli [4] though parity may alter the fate of some of the WAP+ cells [5,6]. Recently, we have reported that a small subset of luminal epithelial cells produce cytokeratin 6a (Krt6a) and are progenitor cells [7].

It has been shown, using the MMTV promoter to express an oncogenic transgene indiscriminately in the luminal epithelial layer, that tumors arise with different latencies depending upon the oncogene [8]. However, these experiments do not provide insight regarding whether these transgenes induced tumors from a specific subset of luminal epithelial cells or not. By breeding transgenic mice expressing *ErbB2* from the MMTV promoter (MMTV-*ErbB2*) with a transgenic mouse line to express the gene encoding the Cre recombinase from the *WAP* promoter (and therefore to mark WAP+ cells), Henry et al. [9] have discovered evidence that WAP+ cells are the cell of origin of tumor initiated by the MMTV-*ErbB2* transgene, which is also supported by Jeselsohn et al. [10]. Others have reported the CD24+Sca1+ progenitor cell subset as the cell of origin for MMTV-*ErbB2*-induced tumors [11,12].

We have reported the use of the avian leukosis virus-derived retroviral vector RCAS for introducing an oncogene into selected mammary luminal epithelial cells that express the gene encoding the TVA receptor, which is normally absent in mammalian cells [13]. We have reported transgenic mice expressing *tva* from the promoter of MMTV (MMTV-*tva*) for indiscriminate infection of the luminal epithelium [13], and from the promoters of WAP, TOP, and Krt6a [7,14–16] for selective infection of WAP+, canonical Wnt signaling-active, and Krt6a+ cells, respectively. Using RCAS carrying a constitutively activated version of *ErbB2* (ca*ErbB2*) to infect WAP-*tva* mice and MMTV-*tva* mice, we have found that the WAP+ luminal cells are more susceptible to tumorigenesis initiated by ca*ErbB2* than the bulk population [15]. This along with the report by Henry et al. [9] strongly suggests that the WAP+ alveolar cell population is especially susceptible to tumorigenesis initiated by ErbB2. Additionally, we have found that the elevated susceptibility of these WAP+ cells to tumorigenesis is likely due to heightened levels of phosphorylated and activated STAT5 [15], which can weaken the apoptosis anticancer barrier that is normally erected by mammary cells following oncogene activation [14].

The natural corollary of the above findings is that that there may be some luminal epithelial cells that are less susceptible or even resistant to transformation by ErbB2. Indeed, we have recently reported that mammary cells that are marked by transcriptional activity of the canonical Wnt signaling-responsive synthetic promoter TOP are “resistant” to transformation by ErbB2—they do not form tumors upon the gain of ca*ErbB2* and appear to have died as a result of ErbB2 activation, possibly a result of the activation of the apoptosis anticancer barrier [16]. Canonical Wnt signaling activity has been associated with mammary cells with properties of stem or progenitor cells [17,18]. Therefore, these data suggest that perhaps the less differentiated mammary cell population marked by Wnt activity may not be the cell of origin for ErbB2-initiated cancer.

To directly test whether the progenitor subset of the luminal epithelium is resistant to ErbB2-initaited tumorigenesis, we injected RCAS-ca*ErbB2* into both Krt6a-*tva* mice and MMTV-*tva* mice and compared their tumor latency. We report here that, unlike the TOP-*tva*-expressing population, the Krt6a population can indeed be induced by ca*ErbB2* to form tumors but is not more susceptible to ca*ErbB2*-initiated tumorigenesis than the bulk mammary epithelial population defined by MMTV. Consequently, they are less susceptible to tumorigenesis than the WAP-positive population.

## Materials and Methods

### Ethics statement

All procedures using mice were performed in compliance with Baylor College of Medicine Animal Care and Use Committee-approved animal protocol (protocol number: AN-2834).

### Transgenic mice and animal care

RCAS virus preparation and mammary intraductal infection have been previously described [1]. Animal care and procedures were approved by the Institutional Animal Care and Use Committee of Baylor College of Medicine and were in accordance with the procedures detailed in the Guide for Care and Use of Laboratory Animals (NIH publication 85–23). The BAC transgenic K6a-*tva* mouse line generation has been described previously [2]. The transgenic MMTV-*tva* mouse line generation has also been described previously [1].

### Virus preparation and delivery to the mammary gland

RCAS virus preparation has been previously described [1]. Mice were deeply anesthesized with Rodent III CCM combination anesthetic DEA-III mixture (0.05 mg/30g body weight) containing ketamine (37.5 mg/ml), xylaxine (1.9 mg/ml), and acepromazine (0.37 mg/ml), administered via intraperitoneal injection. RCAS-*GFP* or RCAS-ca*ErbB2* (10 μL) was delivered into the numbers 2, 3, and 4 mammary gland through intraductal injection into pubertal (5 weeks of age) K6a-*tva* mice and MMTV-*tva* mice. A tracking dye (0.1% bromophenol blue) was used to determine injection success. For RCAS-GFP-infected mice, the infected glands were collected for flow cytometry analysis 2.5–4 days post injection, as previously described [13]. For RCAS-ca*ErbB2*-infected mice, glands were monitored three times per week.

### Tissue processing and immunostaining

Prior to collection of mammary tissue, mice were euthanized via cervical disarticulation while under CO2-induced unconsciousness. Mammary tumors were removed and fixed in 4% paraformaldehyde overnight at 4°C. Fixed tissues were paraffin-embedded, and 3-μm sections were generated. For immunofluorescent staining, deparaffinized slides were incubated with the primary antibodies overnight at 4 degrees Celsius. After three washes with Tris-buffered saline supplemented with 0.05% Tween 20 (TBST), slides were incubated with the fluorophore-conjugated secondary antibodies for 30 minutes at room temperature, washed with Tris-buffered saline supplemented with 0.05% Tween 20, and counterstained with 4′, 6-diamidino-2-phenylindole (DAPI) to identify nuclei.

## Results and Discussion

### A similar rate of viral infection in Krt6a-*tva* and MMTV-*tva* mice is achieved by injecting fewer viral particles in MMTV-*tva* mice

**Fig 1:**
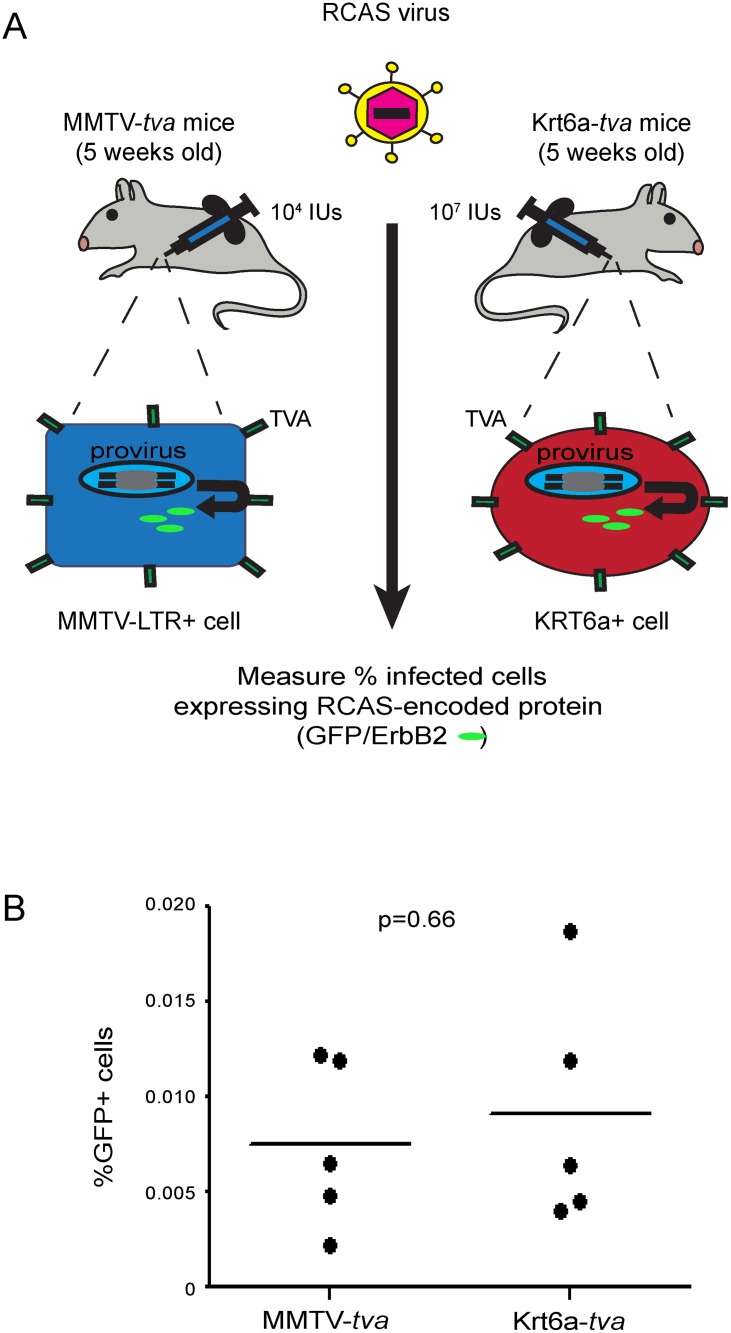
Infection rates are similar between MMTV-*tva* mice and and K6a-*tva* mice after viral dosage adjustment. **(A)** 10^4^ and 10^7^ IUs of RCAS-*GFP* was intraductally injected into the mammary glands of MMTVA-*tva* and K6a-*tva* mice (n = 5). Injected mammary glands were collected 2.5–4 days post-injection to quantify TVA+ cells via flow cytometry. **(B)** The percentage of RCAS-*GFP*-infected cells from K6a-*tva* mice (0.009%) was comparable with that from MMTV-*tva* (0.008%).

To test whether the Krt6a+ cells are at increased or decreased susceptibility to tumorigenesis, it is essential that an oncogene is introduced into the same number of Krt6a+ cells vs. the reference cells. As in the previous studies [7,14–16] comparing the tumorigenetic susceptibility of either WAP+ mammary cells or TOP+ mammary cells with the reference population defined by supporting the promoter activity of MMTV LTR, we also used the intraductal nipple injection of RCAS to introduce genes of interest into target cells. Less than 1% of the luminal cells produce TVA in Krt6a-*tva* mice, while approximately 50% of luminal mammary epithelial cells of MMTV-*tva* make TVA [7,14–16]. Intraductal injection of 1x10^7^ infectious units (IUs) of RCAS carrying the gene encoding green fluorescent protein (RCAS-*GFP*) into 5-week-old Krt6a-*tva* mice led to infection of 0.01% of mammary cells [7]. To ensure comparable rates of oncogene activation in both groups, we titrated the number of viral particles injected into age-matched MMTV-*tva* mice until we also achieved approximate 0.01% of infected cells (Fig. 1).

### Latencies of ca*ErbB2*-induced tumors are similar in Krt6a-*tva* and MMTV-*tva* mice

**Fig 2:**
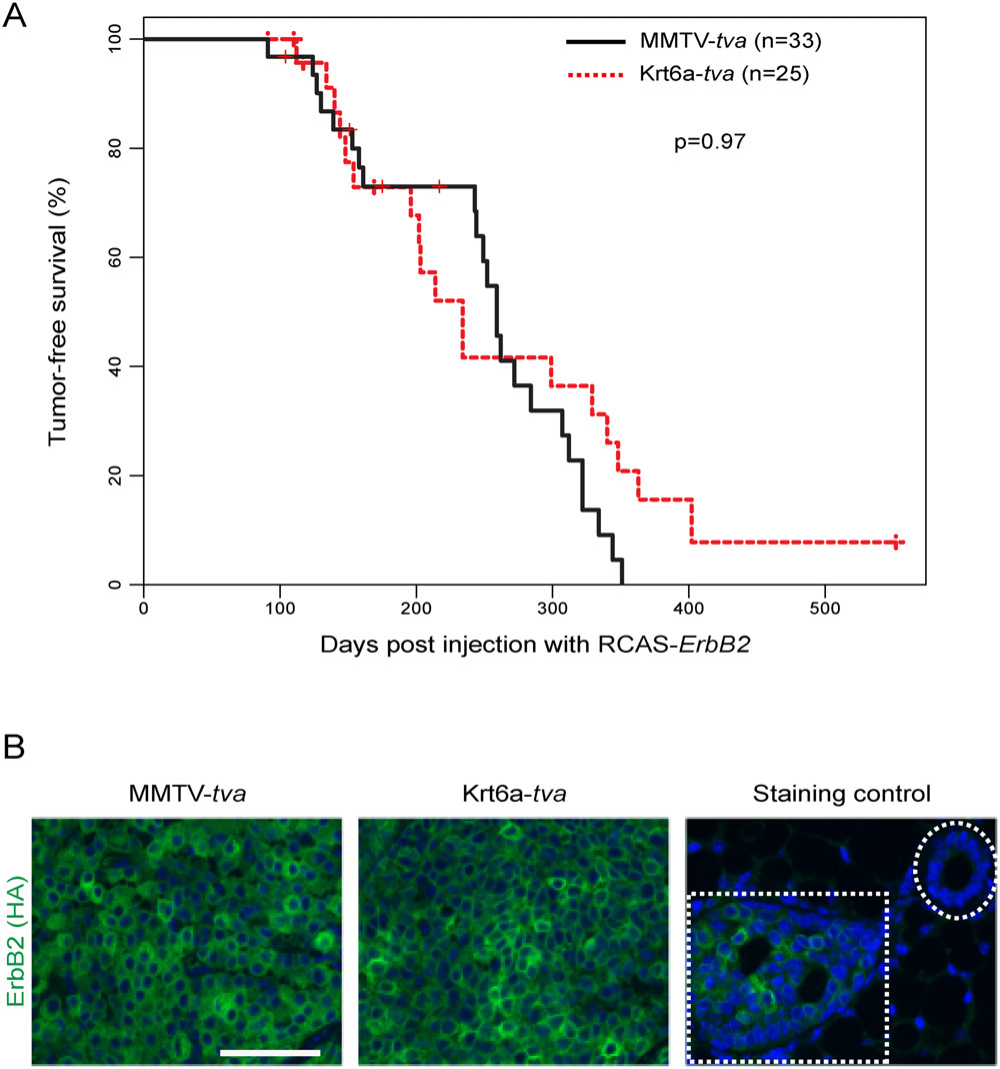
ErbB2 initiated tumors with comparable latency in MMTV-*tva* and K6a-*tva* mice. **(A)** Kaplan-Meier tumor-free survival curves of RCAS-ca*ErbB2*-infected mice of the indicated genotypes. **(B)** Immunofluorescent staining for HA-tagged ErbB2 in tumors from MMTV-*tva* and K6a-*tva* mice (scale bar: 50 um). Antibody specificity was determined by staining early lesions expressing HA-tagged ErbB2 (third panel, bottom left; positive control) and normal ducts (third panel, top right; negative control).

After achieving similar rates of infection in these two lines of *tva* mice, we injected RCAS-ca*ErbB2* into 5-week-old Krt6a-*tva* (n = 25, 1 × 10^7^ IUs) and MMTV-*tva* (n = 33, 1 × 10^4^ IUs) mice and palpated the mice weekly for tumor incidence (defined as a growth ≥2mm). In the MMTV-*tva* cohort, tumors appeared with a median latency of 260 days (Fig. 2A), similar to our previous observation in this line injected with this small number of RCAS-ca*ErbB2* viral particles [16] but dramatically longer than in this line injected with higher doses of this virus [14,19]. In the Krt6a-*tva* cohort, tumors appeared with a median latency of 234 days (Fig. 2A). Kaplan-Meier analysis detected no difference (p = 0.97). Because the median time to tumor detection was long, we confirmed that the tumors were indeed induced by RCAS-ca*ErbB2* based on immunofluorescence staining for the HA-tag in ca*ErbB2* (Fig. 2B). This finding suggests that the Krt6a progenitor population can be induced to form cancer by ca*ErbB2*, but that it is not more susceptible to transformation than the bulk mammary luminal epithelium.

We previously reported that the RCAS-ca*ErbB2* led to tumors more rapidly in WAP-*tva* mice than in MMTV-*tva* mice [14]. This finding suggests that the WAP-positive luminal epithelial population is more susceptible to ca*ErbB2*-induced tumorigenesis than the bulk mammary epithelium. Using the susceptibility of the bulk mammary epithelium to tumor induction by caErbB2 as a reference point, and given that Krt6a+ cells are of equal susceptibility to (Fig. 2A) and WAP+ cells are of greater susceptibility than the reference [14], we infer that Krt6a+ cells are also less susceptible than WAP+ cells to tumor induction by caErbB2.

### ca*ErbB2*-induced premalignant lesions in Krt6a-*tva* and MMTV-*tva* mice display similar levels of pSTAT5 and apoptosis

**Fig 3:**
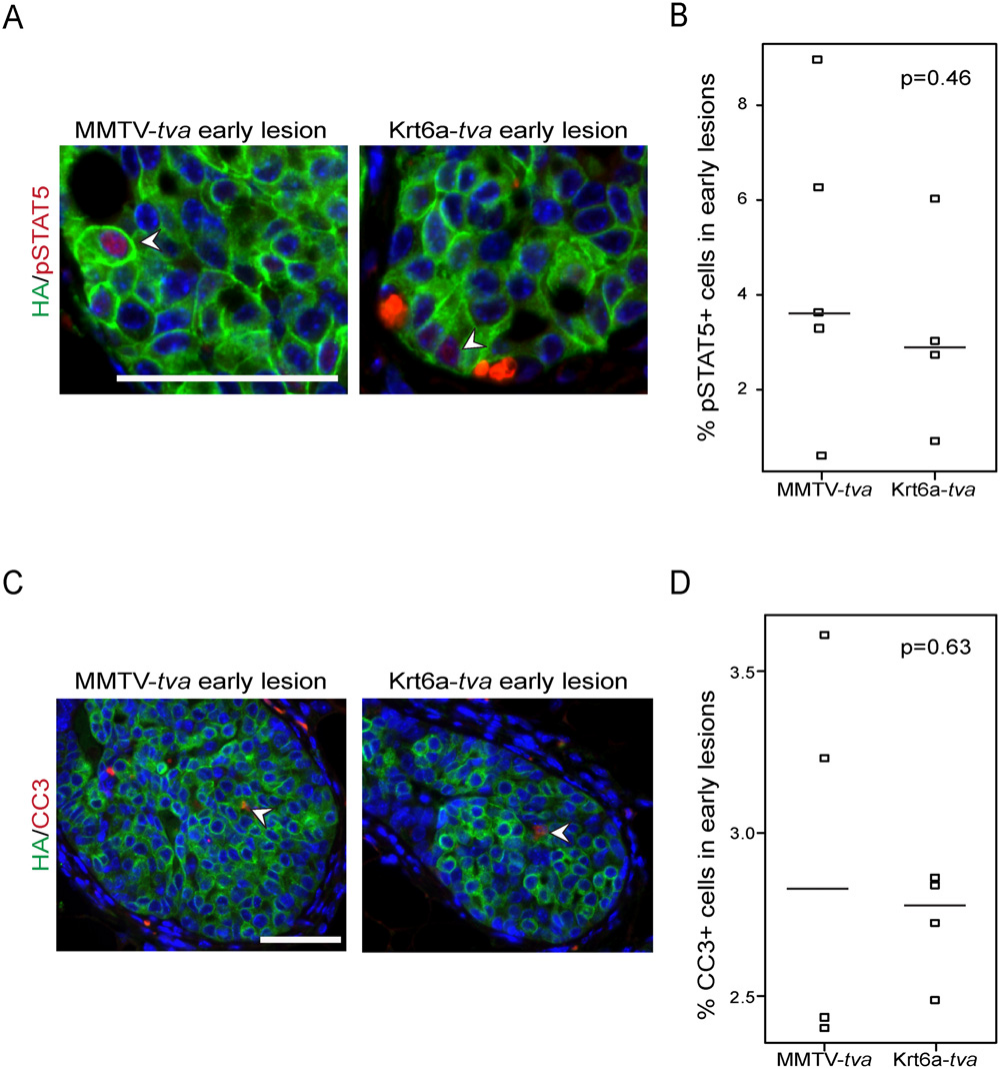
ErbB2-induced premalignant lesions in MMTV-*tva* and K6a-*tva* mice exhibit similar pSTAT5 and apoptosis. **(A)** Co-immunofluorescent staining for HA tagged-ErbB2 (green) and pSTAT5 (red) in tumors from MMTV-*tva* and K6a-*tva* mice, with **(B)** quantification. **(C)** Co-immunofluorescent staining for HA-tagged ErbB2 (green) and cleaved caspase 3 (red) in tumors from MMTV-*tva* and K6a-*tva* mice, with **(D)** quantification (scale bar: 50 um).

STAT5 is a transcriptional factor that is phosphorylated and activated in a subset of mammary epithelial cells, such as those that express WAP [20]. STAT5 activation plays a key role in suppressing apoptosis in mammary early lesions and promoting early lesion progression to cancer [14]. The levels of activated STAT5 (pSTAT5) are higher in early lesions arising from WAP+ cells than from the bulk luminal epithelium defined by MMTV-*tva* expression, and are likely responsible for the accelerated tumorigenesis in WAP-*tva* mice compared to MMTV-*tva* mice [14]. Therefore, we hypothesized that pSTAT5 levels would also be comparable in caErbB2-induced early lesions from Krt6a-*tva* mice versus MMTV-*tva* mice, consistent with their comparable speeds to progress to cancer (Fig. 2A). To measure STAT5 activation in caErbB2-induced early lesion in Krt6a-*tva* mice vs. MMTV-*tva* mice, we used co-immunofluorescence staining for both the HA-tag of ca*ErbB2* and pSTAT5 to determine the percentage of pSTAT5+ cells specifically in caErbB2+ cells in early lesions. We detected pSTAT5 in 3.18% of caErbB2+ cells in early lesions of Krt6a-*tva* mice, a frequency comparable to the 4.56% pSTAT5+ cells detected in early lesions of MMTV-*tva* mice (Fig. 3A, B), but lower than the approximately 22% that we previously detected in early lesions of WAP-*tva* mice [14]. In accordance with this similar low frequency of pSTAT5+ cells, apoptosis was also comparable in early lesions of both Krt6a-*tva* mice and MMTV-*tva* mice (2.7% and 2.8%, respectively; Fig. 3C, D). These levels of apoptosis are similar to that detected previously in RCAS-ca*ErbB2*-induced early lesions in MMTV-*tva* mice [14,21] and were much higher than that in early lesions in WAP-*tva* mice [14]. Collectively, these data suggest that Krt6+ mammary luminal epithelial cells engage the apoptosis anticancer barrier to delay the progression to cancer, in part by limiting STAT5 activation in early lesions.

In conclusion, the cells of origin for breast cancers have been difficult to identify due to a lack of single markers employable in lineage tracing experiments. As an alternative to lineage tracing, we aim to identify potential cells of origin based on the susceptibility of specific mammary epithelial cell populations to transformation by particular oncogenes using the TVA model. To this end, we have generated several mouse models allowing us to target the ErbB2 oncogene into the bulk mammary epithelium, as well as specific, well-defined populations, such as the WAP+, TOP+, and Krt6a+ expressing populations. Using the MMTV model as a baseline, we are thus able to compare (albeit indirectly) the relative susceptibilities of these cell subsets to ErbB2-induced transformation. We found that Krt6a+ progenitor cells can be induced by ErbB2 activation to form tumors, but they are not more vulnerable than the bulk luminal epithelium and appear to be less susceptible than the WAP+ cell subset, possibly due to their failed activation of the STAT5-mediated suppression of the apoptosis anticancer. These findings begin to shed light on the susceptibility of different mammary epithelial populations to ErbB2-induced tumorigenesis, and bring us closer to the identification of the cell of origin of ErbB2+ breast cancer.
